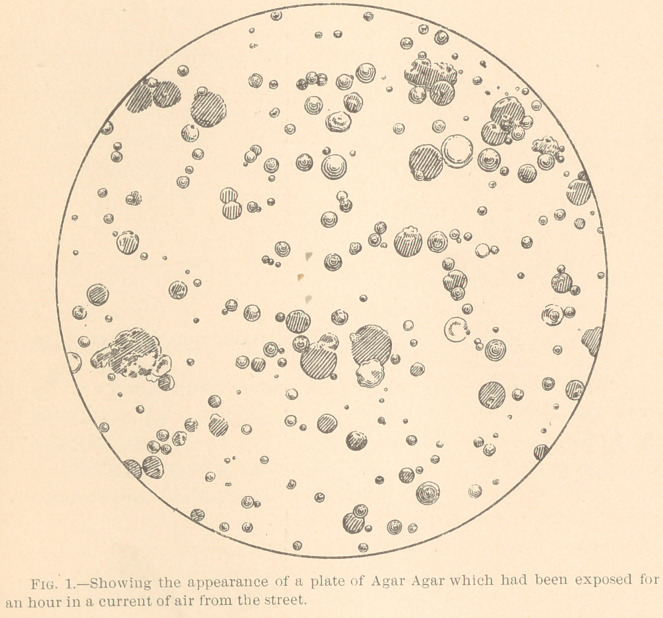# The Bacteria of the Air as a Disturbing Factor in Dental and Surgical Operations

**Published:** 1891-09

**Authors:** W. D. Miller

**Affiliations:** Berlin, Germany


### International Dental Journal.

Vol. XII. September, 1891. No. 9.

### Original Communications.[1]

### THE BACTERIA OF THE AIR AS A DISTURBING FACTOR IN DENTAL AND SURGICAL OPERATIONS.[2]

BY W. D. MILLER, M.D., D.D.S., BERLIN, GERMANY.

The fact that we are surrounded by microscopic organisms always present in the air, water, food, etc., was first definitely established by Ehrenberg in his "Infusionsthierchen als vollkommene Organismen" in 1838, although Leuwenhock had found micro-organisms in the saliva one hundred and fifty years before; and even many centuries earlier, in fact in the first century before Christ, a vague sort of an idea existed that infectious diseases were brought about by an invisible living something, which could be communicated from one person to another through the medium of the air.

At various times and places most fantastic ideas have existed regarding the nature of this agent, and, particularly in the seventeenth century, we are told, at the approach of an epidemic, the inhabitants of the villages assembled *en masse* with kettles, drums, etc., and set up a most fearful din to frighten away the carriers of the disease.

[1] The editor and publishers are not responsible for the views of authors of papers published in this department, nor for any claim to novelty, or otherwise, that may be made by them. No papers will be received for this department that have appeared in any other journal published in the country.

[2] Read before the American Dental Society of Europe, at Heidelberg, August, 1891.

We need not, however, go back to the seventeenth century to meet with notions which disclose just about as great a misconception of the character of the agent under consideration as the prophylactic measures just referred to.

In order to form a proper conception of the distribution, number, and significance of the air-germs it is necessary to acquaint ourselves with their origin, or, in other words, with the manner in which their supply is kept up.

As micro-organisms cannot reproduce themselves in dry air, and for the most part soon die from want of nourishment or moisture, it follows that their number must constantly be recruited to keep up the approximate uniformity observed in the air of any particular locality. It has furthermore been shown by Nägeli and others that bacteria cannot be taken up into the air from liquids undergoing putrefaction or from moist objects, except where the liquid is in a very fine state of division, as foam or spray, in which case it, and of course the bacteria which it contains, may be carried for a short distance by the wind. This source of air-bacteria is of such minor importance that it may practically be left altogether out of account.

Accordingly the chief, and in fact only, source from which the bacteria of the air are recruited is the dried and pulverized organic matter which is caught up and carried from one place to another by the wind in the form of dust.[1]

The air-bacteria are consequently almost invariably found clinging to particles of dust, since any substratum will very rarely be reduced to such a fine state of division as to completely isolate the bacteria which it may contain. It has also been found, as we should naturally expect, that the larger particles of dust carry more bacteria than the smaller ones. The number clinging to each particle will also naturally depend upon the number in the original substratum and upon the resistance which these germs offer to the drying process. Living cholera bacilli, for example, we would not expect to find, because they are killed by drying. In fact many species of bacteria are devitalized by drying, and the majority of all bacteria can withstand the drying process for but a limited time. So that the only means at the disposition of bacteria of finding entrance to the air—*i.e.*, drying—proves to be their greatest enemy, and tends to restrict their numbers.

---

[1] The spores of moulds are not included in this or the following remarks, since we are treating of bacteria only.

The actual number of bacteria in a given quantity of air at any one time will accordingly depend upon the amount of dust in the air and upon the number and kind of bacteria in the substrata from which the dust was formed. As the dust gradually settles in a quiet atmosphere, it follows that the air of a room which has been unoccupied for a few hours will be free from dust and consequently free from germs.

It will be readily seen that the idea conveyed by a quotation in the January number of the INTERNATIONAL DENTAL JOURNAL, page 73, that bacteria exist in the air in swarms something like gnats is entirely erroneous. It may happen that one particle of dust carries one bacterium, and another two or more, but anything like a grouping of dust particles does not occur. Culture plates exposed to the air a sufficient length of time for a number of germs to fall upon them will show a fairly equal distribution of the colonies, as may be seen in Fig. 1.

It was not possible to obtain anything like a definite idea of the number and character of the air-bacteria until the present methods of bacteriological research were introduced, and these have shown that the significance of air-bacteria has been very much overrated, and that the danger of infection by them is not as great as popular notions would lead us to believe. If such were the case, our wonderful modern methods of bacteriological research would be almost useless.

In making a culture on a plate of gelatine or agar-agar, we first take a glass plate from its case and carry it through the air to the levelling apparatus. It is then immediately covered up, but must be exposed again when we pour the gelatine, and a third time when we inoculate the plate, after which it is taken up and carried through the air to the moist chamber. All this may be done, exposing a surface many thousand times as large as that exposed in the opening of a tooth without a single extraneous germ subsequently appearing on the plate.

Again, it has been established that pathogenic bacteria in particular are seldom found in the air, a fact which has been generally recognized by surgeons and has modified surgical methods to a considerable extent.

But a few years ago it was thought indispensable to the success of extensive surgical operations that a spray of carbolic acid or sublimate should be kept going constantly during the operation to prevent an infection by chance air-germs. This practice has now been abandoned, because it has been found that the danger of infection by air-germs is practically null compared with that of infection by unclean hands and instruments.

There is one operation in dental surgery in particular, in which bacteria are supposed to play a most important and disastrous rôle. It is a fact which we were all taught as students, a fact which those of us who are teachers have in turn taught our students, and a fact which we all have learned by sad experience,—that a tooth with a dead pulp, which may never have given any trouble whatever, will develop a severe pericementitis a few hours after the conscientious operator has bored into it and removed the pulp.

This result has been explained on the supposition that germs from the air obtain entrance to the root-canal during the operation, or, as some fancifully put it, are carried into the canal by the air rushing in to fill the vacuum. This being a question of fundamental importance, let us examine it from a purely scientific stand-point and see where we are led to.

In the first place we must do away with the idea that the air "rushes in" when we bore into the pulp-chamber of a tooth containing a putrid pulp; this could not happen unless a partial vacuum existed in the pulp-chamber at the time of operation. But a partial vacuum in the pulp-chamber or root-canal of a tooth with an open apical foramen is an impossibility.

On the contrary, the air, or gas, very frequently rushes out. During the subsequent operation of cleansing the root, however, we grant the possibility of minute quantities of air being introduced. I think I make a very liberal estimate when I grant that ten cubic millimetres of air may be thus carried into the pulp-canal. To be quite sure that we do not underestimate, let us say one hundred cubic millimetres. Again, if we assume that the air of a properly-kept dental office contains four times as many germs as the air of the streets of a large city,—*i.e.*, one germ per litre of air,—I think we are making a liberal estimate. If any one claims that his room contains a larger number of bacteria, we must advise him to keep it in better order.

Now, if one litre of air,—*i.e.*, one million cubic millimetres—contains one germ, how many will one hundred cubic millimetres contain?— $\frac{100}{1000000} = \frac{1}{10000}$.

We could, accordingly, even under the very liberal allowances I have made above, only once in ten thousand times expect to have one germ enter the root-canal. Again, suppose a particle of dust carrying a bacterium should be floating about the opening of the nerve-canal, and actually be drawn into it, if the walls of the canal are moist with some antiseptic, as they should be during the operation, there is no possibility of its being carried far beyond the entrance to the canal before it lodges on the wall, where its passenger will be welcomed with a dose of antiseptic.

How, then, can we account for the " certainty that the opening into a so-called dead tooth would eventuate in pericementitis in a few hours" ? It could at most happen only once in ten thousand times through air-germs ; even then, only, on the supposition that the germ in question arrived alive somewhere near the apex of the root and there found proper conditions for its development. The possibility of particles of dust *falling* into the pulp-chamber during the operation is much greater than that of their being sucked or drawn into it. It may, accordingly, occasionally happen that in this way an air-bacterium gets into the root-canal. This applies, however, only to operations in the lower jaw, since, as we know, germs seldom fall upward. Culture plates turned upside-down may be exposed to the air for some time without much danger of contamination unless strong currents of air are present.

In dental surgery, particularly in the treatment of teeth with necrotic or putrid pulps, there are so many ways in which an infection of the periapical tissue may be brought about that it is not necessary to call into action the one-ten-thousandth of a bacterium, whose acquaintance we have made.

In the simple act of boring into the pulp-chamber we may carry more bacteria into it on the burr than are contained in a whole roomful of air. An unclean nerve-broach inserted into the root-canal may introduce a still greater number of germs.

Again, if in boring into the pulp-chamber we allow the drill to plunge suddenly into it, we are pretty sure to force some of the contents, if they are in a semi-liquid condition, through the foramen ; especially is this the case where the drill is followed up by a burr.

Furthermore, simply wiping out the pulp-chamber with a large pledget of cotton or spunk, forcing a nerve-needle far into the root-canal, especially a needle which nearly fills out the lumen of the canal, or is wound around with cotton so as to act as a piston, may not only force bacteria through the apical foramen, but even particles of the putrefying liquid pulp, by which the infection is invariably seriously complicated.

I call attention in this connection to a series of experiments, recorded in the *Independent Practitioner*, July, 1888, in which mice were infected in a pocket at the root of the tail, some with pieces of gangrenous or putrid pulps, others with pure cultures of bacteria from these pulps. The reaction was invariably much severer in the former case, as might be reasonably expected, since we have the combined mechanical and chemical or toxic irritation in addition to the infection, and in many cases this irritation makes an infection possible where otherwise the tissues would have resisted the invasion of the bacteria.

Again, supposing we have bored into a tooth without having previously sterilized its surface, if, in endeavoring to wipe out the pulp-chamber, we brush over the margin of the drill-hole with our cotton, we may collect large numbers of bacteria and carry them into the pulp-chamber, or, with our cotton, spunk, etc., and still more so with our instruments, we may introduce large numbers of bacteria into the canal, always bearing in mind the fact that we have to deal with a tube which is open at both ends, and in the majority of cases filled with infectious matter often in a semi-fluid condition, we can readily understand how in operating upon one end of the tube we may force some of its contents out at the other unless we carry out our operation with the greatest care and delicacy of manipulation. I have known the taking of an impression with Stent's compound to provoke a severe case of pericementitis simply from the pressure of the material upon the contents of an open pulp-chamber.

In all our operations upon teeth with necrotic or gangrenous pulps we should, above all things, most scrupulously avoid bringing any pressure upon the contents of the root-canal, either by use of too large instruments or by the careless use of cotton in wiping out the cavity, or by suddenly plunging an instrument of any kind into the pulp-chamber or root-canal, or in any of the various other ways in which pressure may be exerted on the contents of the canal.

The tooth operated upon should be as far as possible isolated and cleansed before beginning the operation; no foreign infectious material of any kind should be carried into the canal, either through access of the secretions of the mouth or through unclean instruments, and in particular the cavity of decay, if one is present, should be thoroughly cleansed and disinfected before beginning operations on the root-canal.

During the removal of the pulp, or the remains of the pulp, the pulp-cavity and as far as possible the root-canal should be kept bathed with an antiseptic. The operator should furthermore always bear in mind that the use of antiseptics does not do away with the necessity of exercising the greatest care and delicacy of manipulation in the mechanical cleansing of the root-canal, particularly where the contents of the latter are in a state of putrefaction.

Since the operation of cleansing and filling root-canals has been carried out, I do not believe that among the millions of operations of this nature which have been performed there has been a single one whose success has been in the least degree hazarded by the introduction of air-germs.

## Figures and Tables

**Fig. 1. f1:**